# Crystal structure of bis(μ-{2-[(5-bromo-2-oxido­benzyl­idene)amino]­eth­yl}sulfanido-κ^3^ *N*,*O*,*S*){2,2′-[(3,4-di­thia­hexane-1,6-di­yl)bis­(nitrilo­methanylyl­idene)]bis­(4-bromo­phenolato)-κ^4^ *O*,*N*,*N*′,*O*′}dicobalt(III) di­methyl­formamide monosolvate

**DOI:** 10.1107/S2056989019007217

**Published:** 2019-05-24

**Authors:** Julia A. Rusanova, Vladimir N. Kokozay, Olena Bondarenko

**Affiliations:** a Department of Chemistry, Taras Shevchenko National University of Kyiv, 64/13, Volodymyrska str., Kyiv 01601, Ukraine

**Keywords:** crystal structure, binuclear Co^III^ complex, Schiff bases, 5-bromo­salicyl­aldehyde, cyste­amine (2-amino­ethanthiol)

## Abstract

The title binuclear Co^III^ complex, [Co_2_(C_9_H_8_BrNOS)_2_(C_18_H_16_Br_2_N_2_O_2_S_2_)]·C_3_H_7_NO, with a Schiff base ligand formed *in situ* from cyste­amine (2-amino­ethane­thiol) and 5-bromo­salicyl­aldehyde crystallizes in the space group *P*2_1_. It was found that during the synthesis the ligand undergoes spontaneous oxidation, forming the new ligand H_2_ *L*′ having an S—S bond. Thus, the asymmetric unit consists of one Co_2_(*L*)_2_(*L*′) mol­ecule and one DMF solvent mol­ecule. Each Co^III^ ion has a slightly distorted octa­hedral S_2_N_2_O_2_ coordination geometry. In the crystal, the components are linked into a three-dimensional network by several S⋯ Br, C⋯ Br, C—H⋯Br, short S⋯C (essentially shorter than the sum of the van der Waals radii for the atoms involved) contacts as well by weak C—H⋯O hydrogen bonds. The crystal studied was refined as an inversion twin.

## Abstract 2

The crystal structure of novel binuclear Co^III^ complex with a Schiff base ligand formed *in situ* from cyste­amine (2-amino­ethane­thiol) and 5-bromo­salicyl­aldehyde is reported.

## Chemical context   

Schiff bases represent one of the most widely used organic compounds. The ability to construct novel ligand systems by means of condensation of a variety of readily available aldehydes and amine makes them and their metal complexes ideal candidates for the construction of novel polynuclear compounds as well for investigation of a large range of properties (Mitra *et al.*, 1997 ▸; Bera *et al.*, 1998 ▸; Prabhakaran *et al.*, 2004 ▸; Nesterov *et al.*, 2014 ▸). It has been shown that the formation and cleavage of di­sulfide bonds is important for the biological activity of several sulfur-containing peptides and proteins (Gilbert *et al.*, 1999 ▸; Jacob *et al.*, 2003 ▸), which makes the study of complexes having a multidentate NSO-containing mixed-ligand environment of considerable inter­est. Thus, such complexes can be considered as model objects for studying the active sites of biological systems (Halcrow *et al.*, 1994 ▸). Despite this, very few studies devoted to the synthesis and investigation of complexes of azomethines formed from thio­amino alcohol have been reported. In this work we present a novel binuclear Co^III^ complex with a mixed N,O,S-donor Schiff base ligand derived from the condensation of 5-bromo­salicyl­aldehyde with cyste­amine (2-amino­ethanthiol) hydro­chloride. The synthesis, crystal structure and spectroscopic characterization are described herein.
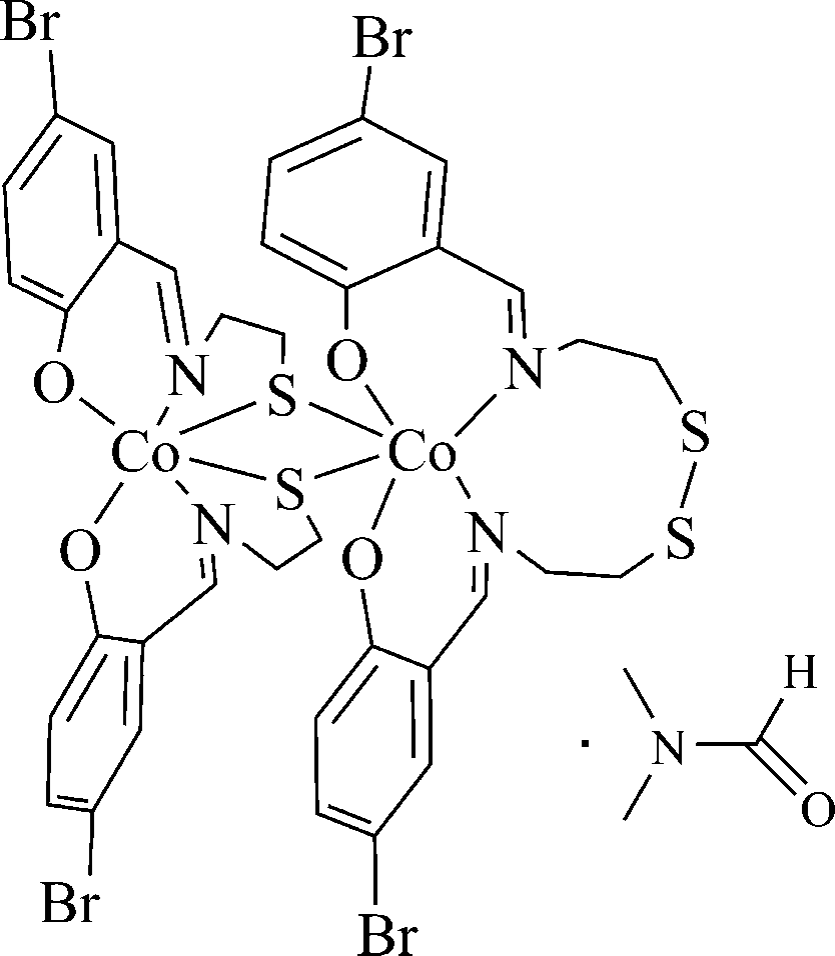


## Structural commentary   

**Figure 1:**
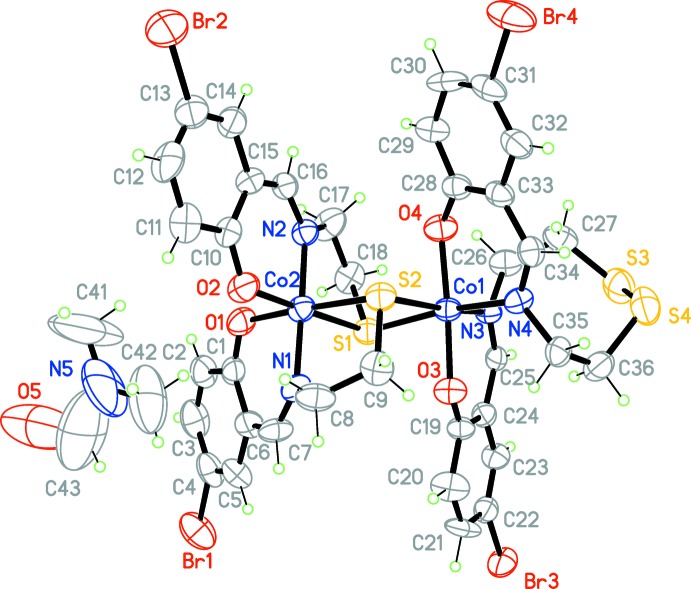
The mol­ecular structure of the title compound, showing 50% probability displacement ellipsoids.

The title compound (Fig. 1 ▸) crystallizes in the monoclinic space group *P*2_1_. The asymmetric unit consists of a binuclear metal complex mol­ecule and a DMF solvent mol­ecule of crystallization. The coordination geometry around each Co^III^ ion can be described as slightly distorted octa­hedral with an S_2_N_2_O_2_ coordination sphere, each ligand spanning the metal atom meridionally. The ligand fragments coordinated to the Co^III^ ions are twisted, as defined by the dihedral angles of 70.41 (2)° between the mean planes of atoms O3/N3/C19/C24/C25 and O4/N4/C28/C33/C34 around Co1, and 64.78 (2)° between the mean planes of atoms O2/N2/C15/C10/C16 and O1/N1/C1/C6/C7 around Co2. During the synthesis, the ligand is partially oxidized with the formation of a –(CH_2_)_2_–S–S–(CH_2_)_2_– bridge. Thus, in contrast to a closely related complex (Chakraborty *et al.*, 1994 ▸), in the title complex the oxidized Schiff base ligand binds to Co1 in a tetra­dentate fashion while the non-oxidized ligand binds to Co2 in a tridentate fashion, and its thiol­ate atoms lie in a *cis*-position, bridging atoms Co1 and Co2. The Co_2_S_2_ bridge is almost planar, with a mean deviation of 0.0673 (4) Å. The Co—S distances in the title complex are in the range 2.207 (3)–2.289 (3) Å, which is generally comparable to the range 2.23–2.26 Å observed for other thio­ether–Co^III^ complexes published earlier (Chakraborty *et al.*, 1994 ▸ and references therein). Contact distances such as for Co⋯Co and S⋯S are also similar.

## Supra­molecular features   

**Figure 2:**
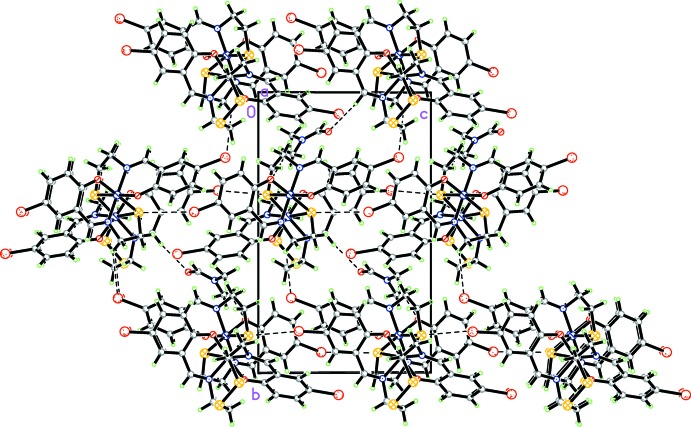
The crystal packing of the title compound viewed along the *a* axis. Weak C—H⋯O hydrogen bonds and C—H⋯Br contacts are shown as dashed lines.

**Figure 3:**
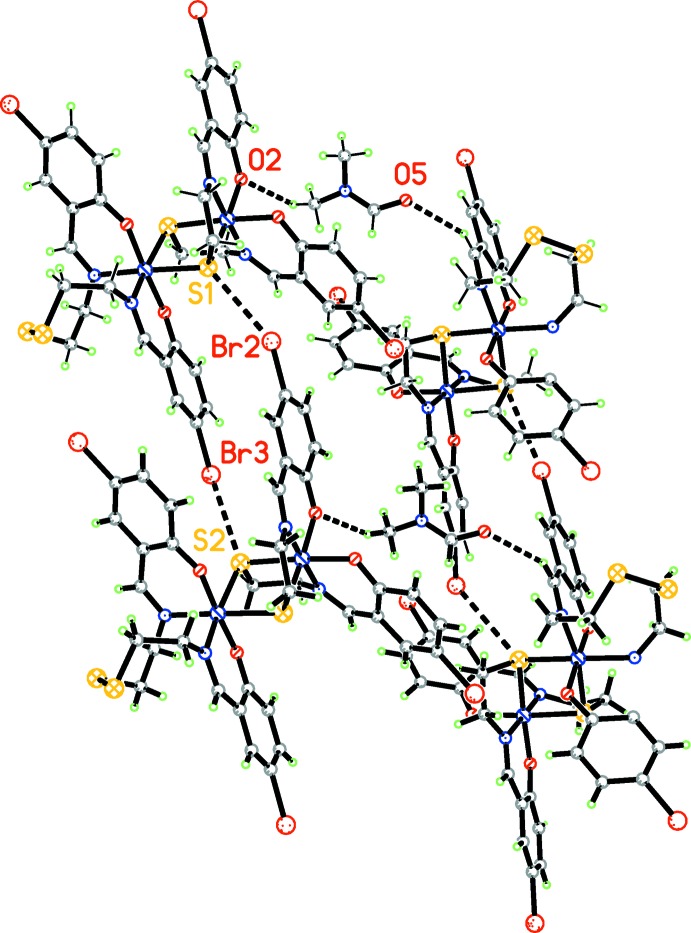
The crystal packing of the title compound with weak C—H⋯O hydrogen bonds and S⋯ Br contacts shown as dashed lines.

**Figure 4:**
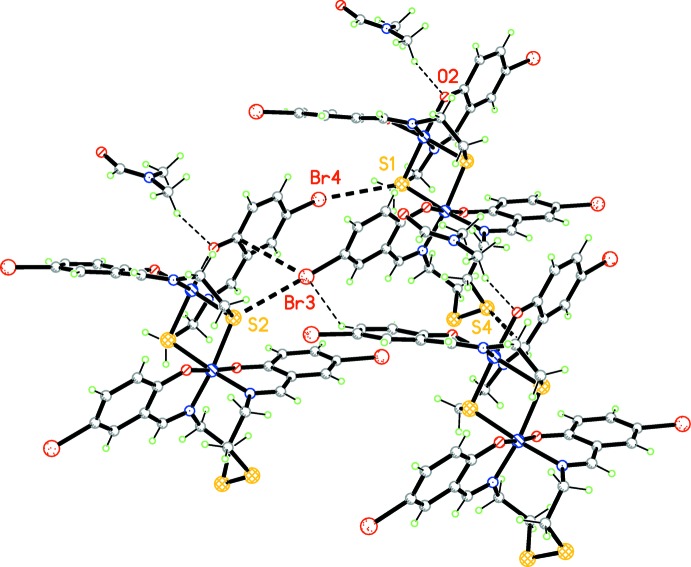
The crystal packing of the title compound. The S⋯Br, C⋯Br and C—H⋯Br contacts that link the components in the crystal are shown as dashed lines.

In the crystal, the bridging ligands are involved in short S⋯Br(*x*, *y*, 1 + *z*) [3.596 (2) Å] and S⋯ Br(*x*, *y*, −1 + *z*) [3.364 (2) Å] contacts, which connect neighboring structural units into chains along [001] (Fig. 2 ▸). The solvent DMF mol­ecules are connected to the complex units by weak C—H⋯O hydrogen bonds (Table 1 ▸, Fig. 3 ▸). In addition, the components are linked by C—H⋯Br (Table 1 ▸), C⋯Br [C10⋯Br = 3 3.443 (8) Å and C15⋯Br3 = 3.506 (7) Å] and short S⋯C contacts. These inter­atomic C⋯Br distances are in agreement with reported data (Echenique-Errandonea *et al.*, 2018 ▸; Tan *et al.*, 2018 ▸). The inter­atomic distances between the aliphatic sulfur atom (S4) and the C16 carbon atom of the ligand of an adjacent mol­ecule (at 1 + *x*, *y*, *z*) are essentially shorter than the sum of the van der Waals radii for the atoms involved [S4⋯C16 = 3.198 (8) Å] (Fig. 4 ▸). Analogous short contacts are well known for coordination compounds with the 1,2,3,4,5-di­thia­diazolyl radical (Beldjoudi *et al.*, 2013 ▸; Boeré, 2016 ▸ and references therein).

## Database survey   

A search of the Cambridge Structural Database (Version 5.40; last update February 2019; Groom *et al.*, 2016 ▸) for related Co complexes with an amino­ethane­thiol group gave 15 hits, including two closely related structures, binuclear bis­[(μ^2^-2-(salicyl­idene­amino)­ethane­thiol­ato]-*N*-(3-thia­pent-5-en­yl)sali­cylaldiminato-*N*,*O*)dicobalt(III) aceto­nitrile solvate and [1,8-bis­(salicyl­idene­amino)-3,6-di­thia­octa­ne)cobalt(III) perchlor­ate with a di­sulfide moiety (Chakraborty *et al.*, 1994 ▸). Closely related structures with short S⋯C contacts are 4-(4-methyl­phen­yl)-3*H*-1,2,3,5-di­thia­diazole (Beldjoudi *et al.*, 2013 ▸) and bis­[4-(4-tri­fluoro­methyl­phen­yl)-1,2,3,5-di­thia­diaz­ol­yl radical] tri­phenyl­stibine (Boeré, 2016 ▸).

## Synthesis and crystallization   

A solution of KOH (0.12 g, 2 mmol) in a minimum amount of methanol was added to a solution of 2-amino­ethanthiol hydro­chloride (0.23g, 2 mmol) in methanol (5 ml) and stirred in an ice bath for 10 min. The white precipitate of solid KCl was removed by filtration and 5-bromo­salicyl­aldehyde (0.40 g, 2 mmol) in di­methyl­formamide (10 ml) were added to the filtrate and stirred on air magnetically for 40 min. Cobalt acetate (0.25 g, 1 mmol) was added to the yellowish solution of the Schiff base formed *in situ*, and the resulting deep-brown solution was stirred magnetically and heated in air at 323–333 K for 2 h. Crystals suitable for X-ray crystallographic study were formed within *ca* 1 month after successive addition of *i*-PrOH into the resulting solution. The crystals were filtered off, washed with dry *i*-PrOH and finally dried at room temperature (yield: 18%). Analysis calculated for C_39_H_39_Br_4_Co_2_N_5_O_5_S_4_ (*M* = 1223.49): C,38.28; N, 5.72; H, 3.21%. Found: C, 38.31; N, 5.79; H, 3.28%. The compound is sparingly soluble in CH_3_CN and good in DMSO, DMF.

The IR spectrum of the title complex in the 4000–400 cm^−1^ range shows the characteristic azomethine group (–H—C=N) peak at 1616 cm^−1^, indicating the formation of the Schiff base. There are no bands assignable to υ(O—H), indicating the loss of the phenolic hydrogen of the free ligand. In addition, all the characteristic functional group peaks are present in the spectrum. Thus, signals in the 3000–3100 cm^−1^ and 1600–1400 cm^−1^ regions were assigned to the aromatic C—H and C—C stretches, and weak bands at 544 cm^−1^ and 684 cm^−1^ to the S—S and C—S stretches, respectively. The very strong bands at 1454 cm^−1^ can be attributed to overlapped C—H bending (scissoring) (in the CH_3_ groups of the solvent mol­ecule) and aromatic –C=C stretching vibrations. Another strong band at 1310 cm^−1^ can be assigned to C—O vibrations.

The structural assignment of the title compound was supplemented by its ^1^H NMR spectra, obtained in DMSO-*d*_6_ at room temperature using TMS as the inter­nal standard. It revealed an azomethine proton singlet at 8.099 ppm as well the increase in spectroscopic complexity in both the aromatic and aliphatic regions. ^1^H NMR, DMSO-*d*_6_, δ in ppm: –CH=N, 8.099 (*s*); aromatic protons (C_6_H_3_): 7.94–6.52; aliphatic protons (–SCH_2_CH_2_N=): 4.44 (*m*); solvent CH_3:_ 2.96 (*s*), 2.8 (*s*). Unfortunately, it could not provide any indication of the dinuclear binding mode, which was revealed only by the X-ray structure determination.

## Refinement   

Crystal data, data collection and structure refinement details are summarized in Table 2 ▸. The hydrogen atoms bonded to carbon were included at geometrically calculated positions (C—H = 0.93–0.97 Å) and refined using a riding model with *U*_iso_(H) = 1.5*U*_eq_(C-meth­yl) and 1.2*U*_eq_(C) for other H atom. The crystal studied was refined as an inversion twin with the ratio of the twin components refining to 0.436 (12):0.564 (12).

## Supplementary Material

Crystal structure: contains datablock(s) I. DOI: 10.1107/S2056989019007217/lh5903sup1.cif

Structure factors: contains datablock(s) I. DOI: 10.1107/S2056989019007217/lh5903Isup2.hkl

CCDC reference: 1916953

Additional supporting information:  crystallographic information; 3D view; checkCIF report

## Figures and Tables

**Table 1:** Hydrogen-bond geometry (Å, °)

*D*—H⋯*A*	*D*—H	H⋯*A*	*D*⋯*A*	*D*—H⋯*A*
C42—H42*B*⋯O2	0.96	2.35	3.25 (2)	157
C25—H25⋯O5^i^	0.93	2.56	3.39 (2)	149
C3—H3⋯Br3^ii^	0.93	2.85	3.633 (13)	142
C17—H17*B*⋯Br1^i^	0.97	3.00	3.743 (11)	134

Symmetry codes: (i) 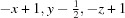; (ii) 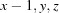.

**Table 2:** Experimental details

Crystal data	
Chemical formula	[Co_2_(C_9_H_8_BrNOS)_2_C_18_H_16_Br_2_N_2_O_2_S_2_)]·C_3_H_7_NO
*M*_r_	1223.49
Crystal system, space group	Monoclinic, *P*2_1_
Temperature (K)	296
*a*, *b*, *c* (Å)	11.532 (3), 17.714 (3), 12.192 (3)
β (°)	116.609 (6)
*V* (Å^3^)	2226.9 (8)
*Z*	2
Radiation type	Mo *K*α
μ (mm^−1^)	4.57
Crystal size (mm)	0.33 × 0.14 × 0.11

Data collection	
Diffractometer	Bruker SMART APEXII
Absorption correction	Numerical face-indexed
*T*_min_, *T*_max_	0.314, 0.633
No. of measured, independent and observed [*I* > 2σ(*I*)] reflections	21805, 8890, 4960
*R*_int_	0.067
(sin θ/λ)_max_ (Å^−1^)	0.630

Refinement	
*R*[*F* ^2^ > 2σ(*F* ^2^)], *wR*(*F* ^2^), *S*	0.051, 0.101, 0.94
No. of reflections	8890
No. of parameters	535
No. of restraints	1
H-atom treatment	H-atom parameters constrained
Δρ_max_, Δρ_min_ (e Å^−3^)	0.45, −0.46
Absolute structure	Refined as an inversion twin
Absolute structure parameter	0.436 (12)

Computer programs: *SMART* and *SAINT* (Bruker, 2008 ▸), *SHELXT* (Sheldrick, 2015*a* ▸), *SHELXL2016/4* (Sheldrick, 2015*b* ▸), *SHELXTL* (Sheldrick, 2008 ▸) and *publCIF* (Westrip, 2010 ▸).

## supplementary crystallographic information

### Crystal data

**Table:**

[Co_2_(C_9_H_8_BrNOS)_2_C_18_H_16_Br_2_N_2_O_2_S_2_)]·C_3_H_7_NO	*F*(000) = 1212
*M**_r_* = 1223.49	*D*_x_ = 1.825 Mg m^−^^3^
Monoclinic, *P*2_1_	Mo *K*α radiation, λ = 0.71073 Å
*a* = 11.532 (3) Å	Cell parameters from 920 reflections
*b* = 17.714 (3) Å	θ = 2.3–18.8°
*c* = 12.192 (3) Å	µ = 4.57 mm^−^^1^
β = 116.609 (6)°	*T* = 296 K
*V* = 2226.9 (8) Å^3^	Prizm, brown
*Z* = 2	0.33 × 0.14 × 0.11 mm

### Data collection

**Table:**

Bruker SMART APEXII diffractometer	8890 independent reflections
Radiation source: sealed tube	4960 reflections with *I* > 2σ(*I*)
Graphite monochromator	*R*_int_ = 0.067
φ and ω scans	θ_max_ = 26.6°, θ_min_ = 1.9°
Absorption correction: numerical face-indexed	*h* = −14→14
*T*_min_ = 0.314, *T*_max_ = 0.633	*k* = −22→22
21805 measured reflections	*l* = −15→13

### Refinement

**Table:**

Refinement on *F*^2^	Hydrogen site location: inferred from neighbouring sites
Least-squares matrix: full	H-atom parameters constrained
*R*[*F*^2^ > 2σ(*F*^2^)] = 0.051	*w* = 1/[σ^2^(*F*_o_^2^) + (0.033*P*)^2^] where *P* = (*F*_o_^2^ + 2*F*_c_^2^)/3
*wR*(*F*^2^) = 0.101	(Δ/σ)_max_ = 0.002
*S* = 0.94	Δρ_max_ = 0.45 e Å^−^^3^
8890 reflections	Δρ_min_ = −0.46 e Å^−^^3^
535 parameters	Absolute structure: Refined as an inversion twin
1 restraint	Absolute structure parameter: 0.436 (12)

### Special details

**Table:**

Geometry. All esds (except the esd in the dihedral angle between two l.s. planes) are estimated using the full covariance matrix. The cell esds are taken into account individually in the estimation of esds in distances, angles and torsion angles; correlations between esds in cell parameters are only used when they are defined by crystal symmetry. An approximate (isotropic) treatment of cell esds is used for estimating esds involving l.s. planes.
Refinement. Refined as a two-component inversion twin

### Fractional atomic coordinates and isotropic or equivalent isotropic displacement parameters (Å^2^)

**Table:**

	*x*	*y*	*z*	*U*_iso_*/*U*_eq_	
BR1	0.27868 (16)	0.76620 (8)	0.18956 (12)	0.0773 (5)	
BR2	0.57579 (17)	0.57341 (10)	1.36338 (12)	0.0919 (6)	
BR3	0.93442 (12)	0.64978 (8)	0.23531 (9)	0.0583 (4)	
BR4	1.20396 (17)	0.42429 (7)	1.46454 (11)	0.0774 (5)	
CO1	0.94686 (14)	0.55635 (7)	0.82744 (11)	0.0349 (4)	
CO2	0.67358 (15)	0.63351 (8)	0.81416 (12)	0.0386 (4)	
S1	0.7359 (3)	0.57089 (15)	0.6932 (2)	0.0408 (7)	
S2	0.8872 (3)	0.63423 (16)	0.9432 (2)	0.0424 (7)	
S3	1.2234 (4)	0.38961 (19)	0.7811 (3)	0.0677 (10)	
S4	1.3413 (4)	0.4697 (2)	0.8932 (3)	0.0722 (11)	
O1	0.4946 (7)	0.6309 (4)	0.6944 (7)	0.050 (2)	
O2	0.6291 (8)	0.6884 (4)	0.9258 (7)	0.049 (2)	
O3	0.9688 (7)	0.6388 (4)	0.7410 (5)	0.0390 (18)	
O4	0.9199 (8)	0.4780 (4)	0.9197 (6)	0.046 (2)	
O5	0.177 (3)	0.8614 (10)	0.602 (2)	0.260 (15)	
N1	0.7010 (9)	0.7301 (5)	0.7595 (8)	0.040 (2)	
N2	0.6434 (9)	0.5376 (4)	0.8673 (7)	0.038 (2)	
N3	0.9625 (8)	0.4804 (5)	0.7185 (7)	0.036 (2)	
N4	1.1350 (8)	0.5635 (5)	0.9465 (7)	0.035 (2)	
N5	0.356 (2)	0.8298 (13)	0.7453 (17)	0.143 (8)	
C1	0.4547 (11)	0.6629 (6)	0.5875 (11)	0.044 (3)	
C2	0.3323 (12)	0.6388 (7)	0.4908 (13)	0.059 (3)	
H2	0.285037	0.601437	0.506436	0.071*	
C3	0.2849 (12)	0.6700 (7)	0.3769 (13)	0.065 (4)	
H3	0.207682	0.651838	0.314888	0.078*	
C4	0.3498 (14)	0.7284 (7)	0.3514 (12)	0.057 (4)	
C5	0.4626 (12)	0.7542 (6)	0.4398 (10)	0.050 (3)	
H5	0.505155	0.793335	0.421852	0.060*	
C6	0.5185 (11)	0.7231 (6)	0.5601 (10)	0.042 (3)	
C7	0.6325 (12)	0.7562 (6)	0.6516 (10)	0.045 (3)	
H7	0.660225	0.801060	0.631401	0.055*	
C8	0.8050 (12)	0.7758 (6)	0.8552 (10)	0.053 (3)	
H8A	0.776427	0.793289	0.914552	0.064*	
H8B	0.823098	0.819709	0.817832	0.064*	
C9	0.9255 (12)	0.7296 (6)	0.9187 (10)	0.051 (3)	
H9A	0.981261	0.752557	0.996978	0.061*	
H9B	0.972097	0.728743	0.869323	0.061*	
C10	0.6155 (11)	0.6597 (6)	1.0181 (9)	0.038 (3)	
C11	0.5939 (11)	0.7097 (7)	1.0972 (10)	0.053 (3)	
H11	0.586732	0.761231	1.081041	0.063*	
C12	0.5833 (13)	0.6823 (8)	1.1993 (11)	0.062 (4)	
H12	0.571389	0.716067	1.251758	0.074*	
C13	0.5899 (13)	0.6072 (8)	1.2237 (10)	0.054 (3)	
C14	0.6085 (11)	0.5577 (7)	1.1483 (9)	0.050 (3)	
H14	0.612356	0.506339	1.165184	0.060*	
C15	0.6221 (10)	0.5824 (6)	1.0453 (8)	0.033 (3)	
C16	0.6296 (11)	0.5264 (6)	0.9648 (10)	0.042 (3)	
H16	0.623739	0.476383	0.984981	0.051*	
C17	0.6270 (13)	0.4697 (6)	0.7917 (10)	0.060 (4)	
H17A	0.541320	0.448716	0.767656	0.071*	
H17B	0.690367	0.432056	0.840066	0.071*	
C18	0.6431 (12)	0.4873 (6)	0.6793 (10)	0.050 (3)	
H18A	0.558072	0.493104	0.610353	0.060*	
H18B	0.685403	0.444890	0.661665	0.060*	
C19	0.9562 (10)	0.6378 (6)	0.6297 (9)	0.034 (2)	
C20	0.9507 (13)	0.7066 (6)	0.5724 (10)	0.052 (3)	
H20	0.951716	0.751035	0.613426	0.062*	
C21	0.9438 (12)	0.7111 (6)	0.4553 (10)	0.052 (3)	
H21	0.940160	0.757612	0.418564	0.062*	
C22	0.9424 (10)	0.6445 (7)	0.3951 (9)	0.042 (3)	
C23	0.9456 (10)	0.5774 (7)	0.4461 (8)	0.041 (3)	
H23	0.943115	0.533509	0.403245	0.049*	
C24	0.9527 (10)	0.5724 (6)	0.5641 (8)	0.038 (3)	
C25	0.9568 (10)	0.4979 (6)	0.6133 (9)	0.036 (3)	
H25	0.955114	0.457710	0.563470	0.043*	
C26	0.9674 (12)	0.3996 (6)	0.7432 (10)	0.047 (3)	
H26A	0.941201	0.372000	0.667030	0.057*	
H26B	0.906448	0.387662	0.775484	0.057*	
C27	1.1019 (12)	0.3743 (6)	0.8341 (11)	0.057 (3)	
H27A	1.099336	0.320973	0.850905	0.068*	
H27B	1.127029	0.401447	0.910528	0.068*	
C28	0.9865 (12)	0.4679 (6)	1.0383 (10)	0.039 (3)	
C29	0.9315 (12)	0.4238 (6)	1.1010 (9)	0.050 (3)	
H29	0.849861	0.402480	1.056680	0.060*	
C30	0.9965 (15)	0.4120 (6)	1.2256 (11)	0.056 (4)	
H30	0.958376	0.383101	1.264395	0.067*	
C31	1.1165 (14)	0.4423 (6)	1.2924 (10)	0.049 (3)	
C32	1.1763 (12)	0.4823 (6)	1.2377 (10)	0.049 (3)	
H32	1.260004	0.500364	1.283822	0.059*	
C33	1.1110 (12)	0.4965 (6)	1.1100 (9)	0.039 (3)	
C34	1.1809 (11)	0.5396 (6)	1.0563 (10)	0.040 (3)	
H34	1.267389	0.550643	1.107427	0.048*	
C35	1.2272 (10)	0.6034 (6)	0.9148 (9)	0.043 (3)	
H35A	1.304458	0.614881	0.989742	0.051*	
H35B	1.188873	0.650857	0.875868	0.051*	
C36	1.2666 (11)	0.5590 (7)	0.8294 (10)	0.054 (3)	
H36A	1.326841	0.589102	0.812178	0.065*	
H36B	1.190367	0.550140	0.752367	0.065*	
C41	0.303 (2)	0.7791 (13)	0.798 (2)	0.238 (18)	
H41A	0.213588	0.770237	0.742278	0.356*	
H41B	0.309157	0.799792	0.873079	0.356*	
H41C	0.349888	0.732326	0.814585	0.356*	
C42	0.4848 (19)	0.8462 (12)	0.8032 (18)	0.142 (9)	
H42A	0.506836	0.877992	0.751540	0.214*	
H42B	0.533990	0.800281	0.820119	0.214*	
H42C	0.504510	0.871953	0.878817	0.214*	
C43	0.284 (4)	0.857 (3)	0.637 (6)	0.33 (4)	
H43	0.321891	0.873753	0.588075	0.400*	

### Atomic displacement parameters (Å^2^)

**Table:**

	*U*^11^	*U*^22^	*U*^33^	*U*^12^	*U*^13^	*U*^23^
BR1	0.0733 (11)	0.0729 (9)	0.0560 (8)	0.0139 (8)	0.0026 (8)	0.0018 (8)
BR2	0.1197 (15)	0.1217 (13)	0.0522 (8)	0.0358 (12)	0.0545 (9)	0.0168 (9)
BR3	0.0510 (8)	0.0923 (10)	0.0329 (6)	0.0013 (8)	0.0199 (6)	0.0129 (7)
BR4	0.1218 (15)	0.0692 (9)	0.0374 (7)	0.0211 (9)	0.0323 (8)	0.0133 (7)
CO1	0.0418 (10)	0.0362 (8)	0.0277 (7)	−0.0014 (8)	0.0165 (7)	0.0009 (7)
CO2	0.0442 (10)	0.0364 (8)	0.0394 (8)	−0.0026 (8)	0.0224 (8)	−0.0026 (7)
S1	0.0432 (19)	0.0482 (18)	0.0303 (14)	0.0017 (15)	0.0158 (14)	0.0006 (14)
S2	0.0497 (19)	0.0478 (17)	0.0300 (13)	−0.0016 (16)	0.0181 (14)	−0.0026 (14)
S3	0.074 (3)	0.061 (2)	0.073 (2)	0.019 (2)	0.037 (2)	0.0007 (19)
S4	0.050 (3)	0.077 (3)	0.081 (2)	0.012 (2)	0.021 (2)	0.009 (2)
O1	0.044 (5)	0.046 (5)	0.065 (5)	−0.010 (4)	0.029 (5)	0.001 (4)
O2	0.067 (6)	0.036 (4)	0.060 (5)	−0.002 (4)	0.042 (5)	−0.005 (4)
O3	0.056 (5)	0.035 (4)	0.026 (4)	−0.008 (4)	0.019 (4)	−0.010 (3)
O4	0.059 (6)	0.048 (5)	0.032 (4)	−0.007 (4)	0.020 (4)	−0.003 (4)
O5	0.53 (5)	0.108 (13)	0.23 (2)	0.05 (2)	0.24 (3)	0.052 (13)
N1	0.040 (6)	0.042 (5)	0.039 (5)	−0.011 (5)	0.018 (5)	−0.007 (5)
N2	0.041 (6)	0.038 (5)	0.036 (5)	−0.001 (4)	0.020 (5)	−0.002 (4)
N3	0.042 (6)	0.035 (5)	0.031 (5)	0.000 (4)	0.018 (5)	0.004 (4)
N4	0.037 (6)	0.035 (5)	0.031 (5)	−0.013 (5)	0.014 (4)	−0.007 (4)
N5	0.105 (19)	0.162 (17)	0.121 (15)	0.064 (15)	0.015 (14)	0.032 (13)
C1	0.037 (8)	0.034 (7)	0.066 (8)	0.002 (6)	0.028 (7)	0.001 (6)
C2	0.036 (8)	0.050 (8)	0.086 (10)	−0.005 (7)	0.021 (8)	0.000 (8)
C3	0.038 (8)	0.067 (10)	0.069 (10)	0.000 (8)	0.004 (8)	−0.015 (8)
C4	0.052 (10)	0.039 (7)	0.067 (9)	0.006 (7)	0.015 (8)	−0.011 (7)
C5	0.051 (9)	0.039 (7)	0.047 (7)	0.006 (7)	0.010 (7)	0.001 (6)
C6	0.042 (8)	0.031 (6)	0.049 (7)	−0.006 (6)	0.017 (7)	−0.005 (6)
C7	0.050 (8)	0.041 (7)	0.041 (7)	−0.017 (6)	0.017 (7)	0.003 (6)
C8	0.069 (10)	0.037 (7)	0.048 (7)	−0.016 (7)	0.021 (7)	−0.001 (6)
C9	0.057 (9)	0.043 (7)	0.050 (7)	−0.003 (6)	0.023 (7)	−0.012 (6)
C10	0.035 (7)	0.043 (7)	0.035 (6)	−0.003 (5)	0.016 (5)	−0.008 (6)
C11	0.044 (9)	0.056 (8)	0.054 (8)	−0.006 (7)	0.017 (7)	−0.019 (7)
C12	0.063 (11)	0.090 (12)	0.042 (8)	0.006 (8)	0.032 (8)	−0.017 (7)
C13	0.058 (10)	0.064 (9)	0.038 (7)	0.005 (7)	0.021 (7)	−0.003 (7)
C14	0.045 (8)	0.062 (8)	0.045 (7)	0.005 (7)	0.022 (6)	0.004 (7)
C15	0.037 (7)	0.037 (7)	0.022 (5)	−0.009 (5)	0.012 (5)	−0.009 (5)
C16	0.033 (7)	0.049 (7)	0.043 (7)	0.002 (6)	0.015 (6)	0.007 (6)
C17	0.079 (11)	0.050 (8)	0.068 (8)	−0.013 (7)	0.050 (8)	−0.019 (7)
C18	0.047 (8)	0.050 (7)	0.048 (7)	−0.015 (6)	0.018 (7)	−0.018 (6)
C19	0.043 (7)	0.028 (6)	0.033 (6)	−0.004 (6)	0.018 (5)	−0.002 (6)
C20	0.072 (10)	0.037 (7)	0.038 (7)	−0.014 (6)	0.016 (7)	−0.014 (6)
C21	0.064 (10)	0.038 (7)	0.040 (7)	−0.006 (7)	0.013 (7)	0.021 (6)
C22	0.034 (7)	0.057 (8)	0.032 (6)	0.003 (6)	0.013 (5)	0.007 (7)
C23	0.050 (8)	0.045 (7)	0.027 (6)	0.001 (6)	0.018 (6)	−0.002 (6)
C24	0.039 (7)	0.044 (7)	0.034 (6)	0.005 (6)	0.019 (6)	0.004 (6)
C25	0.036 (7)	0.040 (7)	0.037 (7)	0.009 (6)	0.021 (6)	0.005 (5)
C26	0.060 (10)	0.040 (7)	0.043 (7)	−0.009 (6)	0.023 (7)	−0.004 (6)
C27	0.065 (10)	0.037 (7)	0.061 (8)	−0.003 (7)	0.022 (8)	0.007 (6)
C28	0.047 (9)	0.032 (6)	0.039 (7)	−0.001 (6)	0.021 (7)	0.000 (6)
C29	0.062 (9)	0.046 (7)	0.040 (7)	0.000 (7)	0.021 (7)	0.009 (6)
C30	0.096 (12)	0.040 (8)	0.048 (8)	−0.004 (8)	0.045 (9)	0.011 (6)
C31	0.082 (11)	0.032 (7)	0.036 (7)	0.024 (7)	0.029 (8)	0.005 (6)
C32	0.061 (9)	0.047 (7)	0.041 (7)	0.019 (7)	0.024 (7)	0.007 (6)
C33	0.052 (9)	0.032 (6)	0.037 (7)	0.010 (6)	0.023 (7)	0.003 (5)
C34	0.036 (7)	0.043 (7)	0.038 (7)	0.000 (6)	0.013 (6)	−0.005 (6)
C35	0.031 (7)	0.045 (7)	0.048 (7)	−0.004 (6)	0.013 (6)	0.002 (6)
C36	0.049 (8)	0.062 (8)	0.058 (7)	−0.006 (7)	0.029 (7)	0.006 (7)
C41	0.14 (2)	0.21 (3)	0.29 (3)	−0.01 (2)	0.03 (2)	0.20 (3)
C42	0.072 (16)	0.21 (2)	0.118 (16)	0.041 (15)	0.014 (13)	−0.031 (15)
C43	0.14 (4)	0.40 (7)	0.50 (9)	0.02 (4)	0.18 (5)	−0.02 (6)

### Geometric parameters (Å, º)

**Table:**

BR1—C4	1.889 (13)	C10—C11	1.411 (14)
BR2—C13	1.880 (12)	C11—C12	1.391 (15)
BR3—C22	1.911 (9)	C11—H11	0.9300
BR4—C31	1.904 (10)	C12—C13	1.358 (16)
CO1—O3	1.883 (6)	C12—H12	0.9300
CO1—O4	1.898 (7)	C13—C14	1.355 (15)
CO1—N3	1.955 (8)	C14—C15	1.403 (13)
CO1—N4	2.003 (9)	C14—H14	0.9300
CO1—S1	2.258 (3)	C15—C16	1.425 (13)
CO1—S2	2.289 (3)	C16—H16	0.9300
CO2—N2	1.905 (8)	C17—C18	1.497 (14)
CO2—N1	1.913 (9)	C17—H17A	0.9700
CO2—O2	1.920 (7)	C17—H17B	0.9700
CO2—O1	1.922 (8)	C18—H18A	0.9700
CO2—S1	2.207 (3)	C18—H18B	0.9700
CO2—S2	2.253 (3)	C19—C20	1.392 (14)
S1—C18	1.790 (11)	C19—C24	1.399 (14)
S2—C9	1.805 (11)	C20—C21	1.396 (14)
S3—C27	1.807 (13)	C20—H20	0.9300
S3—S4	2.016 (5)	C21—C22	1.385 (15)
S4—C36	1.803 (12)	C21—H21	0.9300
O1—C1	1.302 (12)	C22—C23	1.335 (14)
O2—C10	1.306 (11)	C23—C24	1.406 (12)
O3—C19	1.299 (10)	C23—H23	0.9300
O4—C28	1.311 (12)	C24—C25	1.442 (14)
O5—C43	1.11 (5)	C25—H25	0.9300
N1—C7	1.279 (12)	C26—C27	1.515 (15)
N1—C8	1.484 (13)	C26—H26A	0.9700
N2—C16	1.283 (12)	C26—H26B	0.9700
N2—C17	1.475 (12)	C27—H27A	0.9700
N3—C25	1.292 (12)	C27—H27B	0.9700
N3—C26	1.459 (12)	C28—C33	1.399 (15)
N4—C34	1.271 (11)	C28—C29	1.426 (14)
N4—C35	1.465 (12)	C29—C30	1.376 (15)
N5—C43	1.30 (6)	C29—H29	0.9300
N5—C42	1.36 (3)	C30—C31	1.363 (17)
N5—C41	1.39 (3)	C30—H30	0.9300
C1—C6	1.418 (14)	C31—C32	1.355 (16)
C1—C2	1.440 (16)	C32—C33	1.415 (14)
C2—C3	1.361 (16)	C32—H32	0.9300
C2—H2	0.9300	C33—C34	1.461 (14)
C3—C4	1.391 (17)	C34—H34	0.9300
C3—H3	0.9300	C35—C36	1.527 (14)
C4—C5	1.344 (16)	C35—H35A	0.9700
C5—C6	1.422 (14)	C35—H35B	0.9700
C5—H5	0.9300	C36—H36A	0.9700
C6—C7	1.415 (15)	C36—H36B	0.9700
C7—H7	0.9300	C41—H41A	0.9600
C8—C9	1.496 (15)	C41—H41B	0.9600
C8—H8A	0.9700	C41—H41C	0.9600
C8—H8B	0.9700	C42—H42A	0.9600
C9—H9A	0.9700	C42—H42B	0.9600
C9—H9B	0.9700	C42—H42C	0.9600
C10—C15	1.403 (15)	C43—H43	0.9300
			
O3—CO1—O4	176.0 (3)	C13—C14—C15	121.4 (12)
O3—CO1—N3	94.4 (3)	C13—C14—H14	119.3
O4—CO1—N3	89.4 (3)	C15—C14—H14	119.3
O3—CO1—N4	89.0 (3)	C14—C15—C10	119.9 (10)
O4—CO1—N4	91.6 (3)	C14—C15—C16	117.8 (10)
N3—CO1—N4	97.8 (4)	C10—C15—C16	122.0 (9)
O3—CO1—S1	82.9 (2)	N2—C16—C15	127.0 (10)
O4—CO1—S1	95.9 (3)	N2—C16—H16	116.5
N3—CO1—S1	89.1 (3)	C15—C16—H16	116.5
N4—CO1—S1	169.8 (3)	N2—C17—C18	111.7 (9)
O3—CO1—S2	91.7 (2)	N2—C17—H17A	109.3
O4—CO1—S2	84.3 (2)	C18—C17—H17A	109.3
N3—CO1—S2	168.1 (3)	N2—C17—H17B	109.3
N4—CO1—S2	92.4 (2)	C18—C17—H17B	109.3
S1—CO1—S2	81.64 (11)	H17A—C17—H17B	107.9
N2—CO2—N1	179.1 (4)	C17—C18—S1	113.5 (8)
N2—CO2—O2	93.6 (3)	C17—C18—H18A	108.9
N1—CO2—O2	86.2 (3)	S1—C18—H18A	108.9
N2—CO2—O1	86.5 (4)	C17—C18—H18B	108.9
N1—CO2—O1	92.6 (4)	S1—C18—H18B	108.9
O2—CO2—O1	90.8 (3)	H18A—C18—H18B	107.7
N2—CO2—S1	86.7 (3)	O3—C19—C20	118.1 (9)
N1—CO2—S1	93.6 (3)	O3—C19—C24	124.8 (9)
O2—CO2—S1	176.9 (3)	C20—C19—C24	117.1 (8)
O1—CO2—S1	92.3 (2)	C19—C20—C21	122.1 (10)
N2—CO2—S2	94.4 (3)	C19—C20—H20	118.9
N1—CO2—S2	86.5 (3)	C21—C20—H20	118.9
O2—CO2—S2	93.3 (3)	C22—C21—C20	118.4 (9)
O1—CO2—S2	175.7 (2)	C22—C21—H21	120.8
S1—CO2—S2	83.57 (11)	C20—C21—H21	120.8
C18—S1—CO2	96.9 (4)	C23—C22—C21	121.3 (9)
C18—S1—CO1	112.2 (4)	C23—C22—BR3	119.8 (8)
CO2—S1—CO1	98.07 (11)	C21—C22—BR3	118.8 (8)
C9—S2—CO2	99.3 (4)	C22—C23—C24	120.6 (10)
C9—S2—CO1	107.4 (4)	C22—C23—H23	119.7
CO2—S2—CO1	95.88 (11)	C24—C23—H23	119.7
C27—S3—S4	105.0 (4)	C19—C24—C23	120.4 (10)
C36—S4—S3	106.2 (4)	C19—C24—C25	122.2 (8)
C1—O1—CO2	121.8 (7)	C23—C24—C25	117.4 (10)
C10—O2—CO2	125.9 (7)	N3—C25—C24	127.6 (10)
C19—O3—CO1	126.4 (6)	N3—C25—H25	116.2
C28—O4—CO1	125.7 (7)	C24—C25—H25	116.2
C7—N1—C8	121.4 (9)	N3—C26—C27	111.9 (9)
C7—N1—CO2	124.0 (8)	N3—C26—H26A	109.2
C8—N1—CO2	114.5 (7)	C27—C26—H26A	109.2
C16—N2—C17	114.7 (9)	N3—C26—H26B	109.2
C16—N2—CO2	124.8 (8)	C27—C26—H26B	109.2
C17—N2—CO2	120.4 (6)	H26A—C26—H26B	107.9
C25—N3—C26	114.9 (9)	C26—C27—S3	113.4 (8)
C25—N3—CO1	122.0 (7)	C26—C27—H27A	108.9
C26—N3—CO1	122.8 (7)	S3—C27—H27A	108.9
C34—N4—C35	115.4 (9)	C26—C27—H27B	108.9
C34—N4—CO1	123.2 (8)	S3—C27—H27B	108.9
C35—N4—CO1	121.2 (6)	H27A—C27—H27B	107.7
C43—N5—C42	120 (4)	O4—C28—C33	124.8 (10)
C43—N5—C41	120 (4)	O4—C28—C29	119.0 (11)
C42—N5—C41	120.0 (19)	C33—C28—C29	116.2 (10)
O1—C1—C6	125.1 (11)	C30—C29—C28	121.5 (12)
O1—C1—C2	117.9 (10)	C30—C29—H29	119.3
C6—C1—C2	116.9 (11)	C28—C29—H29	119.3
C3—C2—C1	120.9 (12)	C31—C30—C29	120.4 (11)
C3—C2—H2	119.6	C31—C30—H30	119.8
C1—C2—H2	119.6	C29—C30—H30	119.8
C2—C3—C4	121.3 (12)	C32—C31—C30	121.0 (11)
C2—C3—H3	119.4	C32—C31—BR4	119.7 (11)
C4—C3—H3	119.4	C30—C31—BR4	119.2 (10)
C5—C4—C3	119.9 (12)	C31—C32—C33	119.8 (12)
C5—C4—BR1	121.9 (10)	C31—C32—H32	120.1
C3—C4—BR1	118.2 (10)	C33—C32—H32	120.1
C4—C5—C6	121.6 (12)	C28—C33—C32	121.0 (11)
C4—C5—H5	119.2	C28—C33—C34	121.8 (9)
C6—C5—H5	119.2	C32—C33—C34	117.2 (11)
C7—C6—C1	121.5 (10)	N4—C34—C33	126.4 (11)
C7—C6—C5	119.0 (10)	N4—C34—H34	116.8
C1—C6—C5	119.4 (11)	C33—C34—H34	116.8
N1—C7—C6	126.1 (11)	N4—C35—C36	113.9 (8)
N1—C7—H7	117.0	N4—C35—H35A	108.8
C6—C7—H7	117.0	C36—C35—H35A	108.8
N1—C8—C9	110.1 (9)	N4—C35—H35B	108.8
N1—C8—H8A	109.6	C36—C35—H35B	108.8
C9—C8—H8A	109.6	H35A—C35—H35B	107.7
N1—C8—H8B	109.6	C35—C36—S4	112.8 (7)
C9—C8—H8B	109.6	C35—C36—H36A	109.0
H8A—C8—H8B	108.1	S4—C36—H36A	109.0
C8—C9—S2	111.0 (8)	C35—C36—H36B	109.0
C8—C9—H9A	109.4	S4—C36—H36B	109.0
S2—C9—H9A	109.4	H36A—C36—H36B	107.8
C8—C9—H9B	109.4	N5—C41—H41A	109.5
S2—C9—H9B	109.4	N5—C41—H41B	109.5
H9A—C9—H9B	108.0	H41A—C41—H41B	109.5
O2—C10—C15	124.6 (9)	N5—C41—H41C	109.5
O2—C10—C11	118.1 (10)	H41A—C41—H41C	109.5
C15—C10—C11	117.3 (10)	H41B—C41—H41C	109.5
C12—C11—C10	120.4 (12)	N5—C42—H42A	109.5
C12—C11—H11	119.8	N5—C42—H42B	109.5
C10—C11—H11	119.8	H42A—C42—H42B	109.5
C13—C12—C11	121.3 (11)	N5—C42—H42C	109.5
C13—C12—H12	119.4	H42A—C42—H42C	109.5
C11—C12—H12	119.4	H42B—C42—H42C	109.5
C14—C13—C12	119.7 (11)	O5—C43—N5	121 (6)
C14—C13—BR2	120.9 (10)	O5—C43—H43	119.7
C12—C13—BR2	119.4 (9)	N5—C43—H43	119.7

### Hydrogen-bond geometry (Å, º)

**Table:**

*D*—H···*A*	*D*—H	H···*A*	*D*···*A*	*D*—H···*A*
C42—H42*B*···O2	0.96	2.35	3.25 (2)	157
C25—H25···O5^i^	0.93	2.56	3.39 (2)	149
C3—H3···Br3^ii^	0.93	2.85	3.633 (13)	142
C17—H17*B*···Br1^i^	0.97	3.00	3.743 (11)	134

Symmetry codes: (i) −*x*+1, *y*−1/2, −*z*+1; (ii) *x*−1, *y*, *z*.
